# Unified-Removal: A Semi-Supervised Framework for Simultaneously Addressing Multiple Degradations in Real-World Images †

**DOI:** 10.3390/jimaging11110405

**Published:** 2025-11-11

**Authors:** Yongheng Zhang

**Affiliations:** State Key Laboratory of Networking and Switching Technology, BUPT, No. 10 Xitucheng Road, Haidian District, Beijing 100876, China; zhangyongheng@bupt.edu.cn

**Keywords:** multiple degradations removal, real-world image enhancement, knowledge distillation, contrastive learning

## Abstract

This work introduces Uni-Removal, an innovative two-stage framework that effectively addresses the critical challenge of domain adaptation in unified image restoration. Contemporary approaches often face significant performance degradation when transitioning from synthetic training environments to complex real-world scenarios due to the substantial domain discrepancy. Our proposed solution establishes a comprehensive pipeline that systematically bridges this gap through dual-phase representation learning. In the first stage, we implement a structured multi-teacher knowledge distillation mechanism that enables a unified student architecture to assimilate and integrate specialized expertise from multiple pre-trained degradation-specific networks. This knowledge transfer is rigorously regularized by our novel Instance-Grained Contrastive Learning (IGCL) objective, which explicitly enforces representation consistency across both feature hierarchies and image spaces. The second stage introduces a groundbreaking output distribution calibration methodology that employs Cluster-Grained Contrastive Learning (CGCL) to adversarially align the restored outputs with authentic real-world image characteristics, effectively embedding the student model within the natural image manifold without requiring paired supervision. Comprehensive experimental validation demonstrates Uni-Removal’s superior performance across multiple real-world degradation tasks including dehazing, deraining, and deblurring, where it consistently surpasses existing state-of-the-art methods. The framework’s exceptional generalization capability is further evidenced by its competitive denoising performance on the SIDD benchmark and, more significantly, by delivering a substantial 4.36 mAP improvement in downstream object detection tasks, unequivocally establishing its practical utility as a robust pre-processing component for advanced computer vision systems.

## 1. Introduction

The reliability of modern vision systems—from autonomous vehicles to surveillance networks—heavily depends on the quality of their visual inputs. However, these systems frequently operate in uncontrolled environments where images are compromised by diverse degradations including haze, rain, and blur. While humans can intuitively separate these artifacts from scene content, computational models face a fundamental challenge: each degradation type follows distinct physical models, yet a practical system must handle them all without prior knowledge of the specific degradation present.

**Figure 1:**
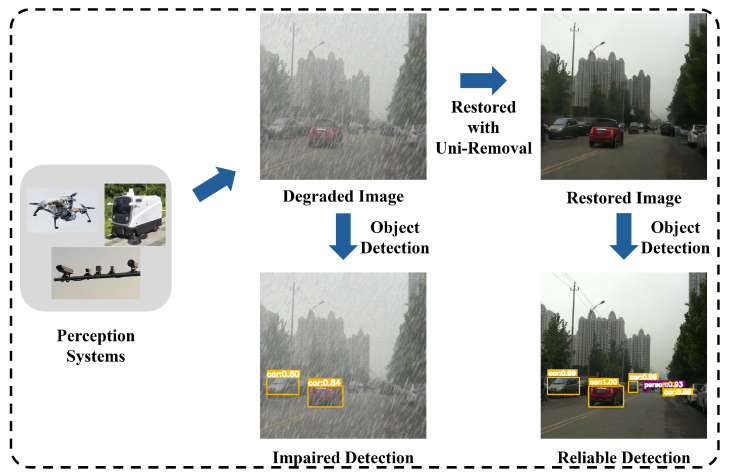
The impact of image degradation in real-world perception systems.

As demonstrated in Figure 1, these degradations not only reduce perceptual quality but critically impair high-level vision tasks. For instance, haze-induced contrast loss can reduce object detection accuracy by over 4% mAP, while blur directly impacts tracking reliability in autonomous systems.

Current approaches to this multi-faceted problem fall into three categories, each with distinct limitations. Task-Specific Deep Models, including prior-based models [1,2,3,4,5,6,7,8,9] and end-to-end architectures [10,11,12,13,14,15,16,17,18], have achieved remarkable success by learning to map from degraded to clean images for a single type of corruption. However, their specialization is their primary weakness—they cannot generalize to unseen or compound degradations. Architectures [19,20,21,22,23,24] represent a step towards generality, handling multiple degradations within a single network framework. Yet, this often comes at the cost of efficiency, as they may require switching specialized parameters or encoders for different inputs. Most relevantly, emerging All-in-One Methods [25,26,27,28] push this further by employing a shared set of parameters for all degradations. However, they remain fundamentally constrained by the synthetic–real domain gap, as their training typically relies on artificially generated degradations that poorly match the complex statistics of real-world image corruptions.

This landscape exposes the dual challenges our work addresses: (1) achieving genuine parameter efficiency and unified handling without performance compromise across degradation types, and (2) closing the substantial domain gap between synthetic training environments and real-world deployment scenarios.

We introduce Uni-Removal, a framework that reframes multi-degradation restoration as a sequential knowledge consolidation problem. Rather than treating different degradations as separate tasks, our method establishes a continuous learning pathway from specialized expertise to generalized real-world competence.

The Knowledge Transfer Stage is architected as a multi-teacher refinement process, where a unified student network learns to integrate removal capabilities for different degradations. Our key innovation here is the Instance-Grained Contrastive Learning (IGCL) loss, which establishes a dual-alignment objective: it enforces consistency between student and teacher outputs at both the feature level, preserving structural representations, and the image level, maintaining perceptual integrity. This ensures the student network learns a robust and generalized restoration capability that transcends the expertise of any single teacher.

Subsequently, the Domain Adaptation Stage functions as a semantic-aware fine-tuning phase, designed to bridge the synthetic-to-real gap. We deploy a Cluster-Grained Contrastive Learning (CGCL) loss in conjunction with adversarial training. The CGCL operates by projecting real-world degraded images and clear images into a shared semantic space, organizing them into clusters based on content similarity rather than degradation type. This enables the model to discern that, for instance, a haze-obscured car and a clear-weather car are semantically closer than two haze images of different objects. This semantic clustering, refined by the adversarial discriminator, guides the student network to generate outputs that are not only visually realistic but also semantically congruent with the manifold of natural images.

In summary, this work makes three key contributions:

- A Synergistic Architecture for Unified Restoration: We propose a coherent two-phase framework that effectively reconciles the conflict between specialized knowledge acquisition and general-purpose application. This is achieved by sequentially integrating multi-teacher distillation with semantic-aware domain adaptation.
- Multi-Granularity Contrastive Learning: We introduce dual contrastive losses that enforce consistency at complementary levels. The IGCL ensures precise sample-wise alignment during knowledge transfer, while the CGCL performs distribution-level alignment during domain adaptation, collectively enhancing the robustness of the restored outputs.
- Extensive Validation of Practical Utility: Beyond state-of-the-art performance on standard tasks (dehazing, deraining, deblurring), we demonstrate the model’s practical value through its compelling cross-task denoising capability and, most importantly, its measurable positive impact on the accuracy of a downstream object detection system.

## 2. Related Work

In this section, we provide a brief overview of the research related to single degradation removal methods, multi-degradation removal methods, knowledge distillation, and contrastive learning, which are relevant to our work.

### 2.1. Single Degradation Removal

#### 2.1.1. Supervised Methods

Over time, the performance of methods based on priors [1,2,3,4,5,6] become increasingly unsatisfactory. The advent of deep convolutional neural networks (CNNs) and the availability of large-scale synthetic datasets have led to an increased interest in learning-based methods for single degradation removal. Early approaches [29,30,31] focused on estimating parameters in physical scattering models using neural networks.

Subsequently, various end-to-end methods [11,12,13,14,15,16,17,32,33] have been proposed to directly restore clear images without relying on explicit physical scattering models. For instance, Zhang et al. [12] introduced a fish retina-inspired dehazing method that incorporates special retinal mechanisms to extract wavelength-dependent degradation information. Hao et al. [15] addressed the challenge of size mismatch between rain streaks during the training and testing phases by employing a monogenic wavelet transform-like hierarchy and a self-calibrated dual attention mechanism. Li et al. [33] incorporated depth information into CNN-based models for dynamic scene deblurring. Additionally, Li et al. [17] proposed DehazeFlow, the first work to utilize normalizing flows for single image dehazing.

#### 2.1.2. Semi-Supervised and Unsupervised Methods

Domain adaptation aims to bridge the gap between a source domain and a target domain. Existing approaches [34,35,36] aim to align the source and target domains either at the feature level or pixel level by minimizing designed losses.

For instance, Kupyn et al. [37] proposed DeblurGAN-V2, a framework based on a relativistic conditional generative adversarial network (GAN) with a double-scale discriminator. They also introduced the feature pyramid network into deblurring. Shao et al. [38] presented a domain adaptation framework that incorporates real hazy images into the training process using a cycle-GAN. Similarly, Wei et al. [39] employed a similar structure in image deraining and introduced a rain attention mechanism. Chen et al. [40] explored a range of physical priors and developed a loss committee to guide the training on real hazy images.

While these methods demonstrate remarkable generalization performances for specific degradations, they often experience significant performance degradation when applied to other types of degradations. In contrast, our proposed Uni-Removal framework effectively addresses the removal of multiple real-world degradations simultaneously.

### 2.2. Multi-Degradation Removal

#### 2.2.1. Multi-Tasks Methods

Furthermore, several studies have explored the use of unified models to address multiple degradation removal problems [21,22,23,24,41]. For instance, Pan et al. [42] proposed DualCNN, which incorporates two parallel branches to recover structures and details in an end-to-end manner. Zhang et al. [21] designed a residual dense network specifically for image restoration. Zamir et al. [22] adopted a multi-stage approach to restore degraded images and introduced an innovative per-pixel adaptive design that leverages in-situ supervised attention to reweight local features at each stage. In addition, Mao et al. [24] introduced an idempotent constraint into the deblurring framework, allowing the framework to be utilized for dehazing and deraining tasks as well.

These methods have demonstrated remarkable results across various degradation types using a unified framework. However, they typically require different sets of pre-trained weights for each type of degradation.

#### 2.2.2. All-in-One Degradations Removal

Li et al. [25] proposed an end-to-end network with multiple encoders and a shared decoder, referred to as the All-in-One network. This network incorporates a discriminator to simultaneously assess the correctness and classify the degradation type of the enhanced images. Furthermore, an adversarial learning scheme is employed, wherein the loss of a specific degradation type is only backpropagated to the corresponding task-specific encoder.

Valanarasua et al. [26] developed a transformer-based end-to-end model consisting of a single encoder and a decoder. Specifically, the TransWeather model utilizes a novel transformer encoder with intra-patch transformer blocks to enhance attention within patches, along with a transformer decoder that incorporates learnable weather type embeddings to adapt to the specific weather degradation.

Chen et al. [27] adopted a two-stage knowledge learning process, which includes knowledge collation and knowledge examination, for adverse weather removal. In the collation stage, a collaborative knowledge transfer technique is proposed to guide the student model in integrating and learning the knowledge of various weather types from well-trained teacher models. In the examination stage, a multi-contrastive regularization approach is adopted to enhance the robustness of the student network for comprehensive weather removal.

As summarized in Table 1, while existing All-in-One methods have made significant progress in unifying models for multiple degradations, they predominantly rely on synthetic data and often require task-specific parameter sets or complex architectures. This limits their efficacy in real-world scenarios. Our proposed Uni-Removal framework, in contrast, is designed to address these limitations by employing a two-stage semi-supervised approach that enables the use of a single set of parameters while effectively bridging the synthetic-to-real domain gap.

### 2.3. Knowledge Distillation

Knowledge distillation [43] originally aimed to transfer knowledge from a large teacher model to a smaller student network. This involved training the student network on a transfer set while leveraging the soft target distribution provided by the larger model. However, it has been shown by Adriana et al. [44] that the teacher network does not necessarily have to be larger than the student network. In fact, both the outputs and intermediate representations learned by the teacher can enhance the training process and improve the final performance of the student network.

The concept of knowledge distillation has found wide application in various high-level computer vision tasks, including object detection [45], face recognition [46], and semantic segmentation [47]. More recently, researchers have also integrated knowledge distillation into image enhancement tasks [27,48].

In contrast to traditional knowledge distillation methods that solely learn from positive examples provided by the teacher network, we propose a MGCL loss in the knowledge transfer stage to enable learning from both positive and negative examples.

### 2.4. Contrastive Learning

Contrastive learning is a technique that aims to sample positive and negative pairs from a given anchor point and then applies different contrastive losses to attract positive samples and repel negative samples [49,50,51,52,53,54].

In recent years, contrastive learning has been introduced into low-level vision tasks such as image-to-image translation [55], deraining [56,57], and dehazing [58,59]. While these methods demonstrate the versatility of contrastive learning, their application paradigms are often directly adapted from high-level vision tasks. For instance, Chen et al. [58] proposed an unsupervised contrastive CDD-GAN framework based on CycleGAN [60] for image dehazing, where positive and negative samples are sampled from the hazy domain and clear domain, respectively. Similarly, Ye et al. [56] devised a novel non-local contrastive learning mechanism that leverages the inherent self-similarity property for image deraining.

However, a key challenge in applying contrastive learning to image restoration remains: how to construct semantically meaningful positive and negative pairs without high-level semantic labels. Most existing approaches in this domain operate within a single stage (e.g., unsupervised domain translation) and do not explicitly address the dual challenges of multi-task knowledge transfer and synthetic-to-real domain adaptation simultaneously. As we will illustrate in Table 2, our work differentiates itself by proposing two dedicated contrastive losses tailored for these specific challenges within a unified, two-stage framework.

## 3. Proposed Method

In this section, we present a normative definition of the task and provide an overview of our proposed method, including its underlying idea and overall structure. Additionally, we delve into the two training stages in detail and conclude with an introduction to the loss functions employed.

### 3.1. Overview

Real-world image restoration demands a framework that achieves two synergistic objectives: mastering the removal of distinct degradations through supervised learning, and adapting this capability to authentic imagery where ground truth is unavailable. The Uni-Removal framework meets this demand through a purpose-built, sequential architecture comprising a Knowledge Transfer Stage followed by a Domain Adaptation Stage.

This design is motivated by the observation that while learning from synthetic data provides a strong foundational model, a critical performance gap remains when deploying in real-world scenarios due to domain shift. Our framework explicitly addresses this lifecycle of model development and deployment, transitioning from knowledge acquisition to practical application. The sequential nature of this approach ensures that the model first establishes robust restoration principles in a controlled synthetic environment before learning to apply them to the complexities of real-world imagery.

**Figure 2:**
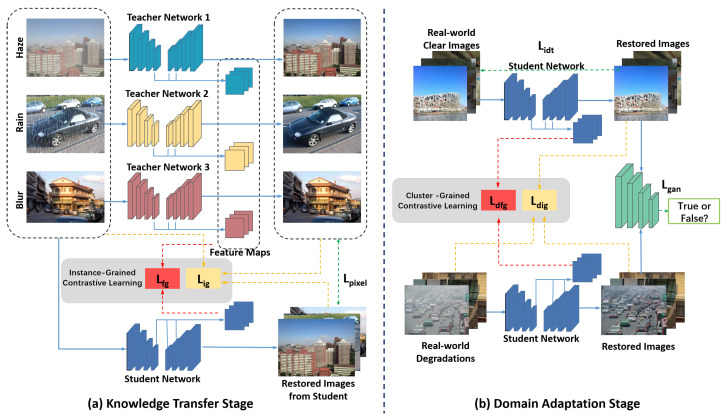
Framework of the proposed Uni-Removal. (**a**) Knowledge Transfer Stage: The student network is guided by multiple well-trained teacher networks (trained on diverse synthetic datasets), utilizing a pixel-level L1 loss ($L_{pixel}$), a feature-level IGCL loss ($L_{fg}$), and an image-level IGCL loss ($L_{ig}$). (**b**) Domain Adaptation Stage: The student network acts as a generator, trained adversarially with a discriminator. The discriminator aims to distinguish between image generated from clear image and image generated from degraded image, while the student is trained to fool it using an adversarial loss ($L_{gan}$). Additional constraints include an identity loss ($L_{idt}$), a feature-level CGCL loss ($L_{dfg}$), and an image-level CGCL loss ($L_{dig}$).

Formally, the model is trained on synthetic datasets $D_{S}={\left\{X_{S_{i}},Y_{S_{i}}\right\}}_{i=1}^{k}$ and real-world datasets $D_{R}={\left\{X_{R_{i}}\right\}}_{i=1}^{k}\cup X_{C}$. As illustrated in Figure 2, the Knowledge Transfer Stage cultivates a versatile student network by distilling expertise from an ensemble of pre-trained, degradation-specific teachers, establishing a comprehensive understanding of diverse degradation patterns. The subsequent Domain Adaptation Stage then refines this student network, calibrating its outputs to align with the statistical distribution of real-world clear images through an adversarial training paradigm that bridges the synthetic–real domain gap. This two-stage progression ensures the model is both technically proficient and practically viable for real-world deployment.

### 3.2. Knowledge Transfer Stage

The primary goal of this stage is to equip a single student network with the collective ability to address multiple degradation types, a challenge that is more effectively met by learning from specialized experts than through direct training on a mixed dataset. This approach circumvents the common issue of performance interference that often plagues models trained simultaneously on heterogeneous restoration tasks.

We first independently train a set of teacher networks ${\left\{G_{T_{i}}\right\}}_{i=1}^{k}$, each specializing in a specific degradation type using its respective synthetic dataset $\left(X_{S_{i}},Y_{S_{i}}\right)$. These teachers share an identical architecture (Multi-stage Progressive Restoration Network, MPR-Net) but possess distinct parameters optimized for their dedicated task, allowing them to develop deep, specialized knowledge of their assigned degradation characteristics and removal strategies.

The student network $G_{S}$, which shares the teacher’s architecture, is then trained to emulate the teachers’ restoration capabilities through a carefully designed knowledge distillation process. The learning signal for the student is derived from a composite objective function that evaluates the student’s output against the teachers’ outputs. This includes a foundational pixel-wise reconstruction loss that ensures basic fidelity, and our proposed Instance-Grained Contrastive Learning (IGCL) objective. The IGCL is engineered to ensure that the student network internalizes the teachers’ restoration strategies at multiple levels of representation, fostering a deeper understanding beyond superficial output matching and enabling more robust generalization.

**Figure 3:**
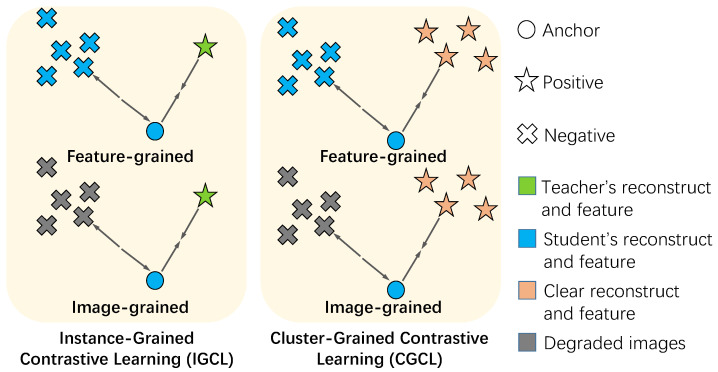
Interpretation of the proposed Instance-Grained Contrastive Learning (IGCL) loss and Cluster-Grained Contrastive Learning (CGCL) loss. IGCL selects one strongly correlated positive and multiple negatives for each sample. However, since a strongly correlated positive sample is not readily available in real-world degradation removal, the CGCL replaces the strongly correlated positive with a batch of weakly correlated positives. This adaptation allows for a more effective learning process in the absence of strongly correlated positive samples in real-world scenarios.

As illustrated in Figure 3, the IGCL comprises a feature-grained contrastive loss and an image-grained contrastive loss. In the feature-grained contrastive loss, the intermediate features of the corresponding teacher network are treated as positives in relation to the intermediate features of the student network, while a batch of intermediate features from the student network with different degradation types are considered as negatives. In the image-grained contrastive loss, the restored image from the corresponding teacher network is regarded as positive with respect to the restored image from the student network, while a batch of synthetic images with various degradations serve as negatives.

Upon completion of this stage, the student network $G_{S}$ becomes a capable unified model for restoring synthetically degraded images, having integrated the diverse expertise of all specialized teachers. However, its performance on real-world images remains limited due to the domain distribution mismatch, necessitating the next stage of training to bridge this gap.

### 3.3. Domain Adaptation Stage

Building upon the foundation established in the knowledge transfer stage, the domain adaptation stage addresses the critical challenge of transferring the student network’s synthetic-domain expertise to authentic real-world imagery. This phase is designed to recalibrate the model’s output space, ensuring that restored images not only achieve technical correctness but also exhibit the nuanced visual characteristics of natural images.

The adaptation process employs a multi-faceted learning strategy centered around distributional alignment. At its core, an adversarial training scheme pits the student network against a discriminator, creating a dynamic where the student learns to produce outputs that are statistically indistinguishable from real clear images. This adversarial objective drives the model to capture the subtle textures, color distributions, and noise patterns that define authentic imagery, moving beyond the sometimes artificial appearance of synthetically trained models.

To maintain the integrity of already-clear inputs, we incorporate an identity preservation constraint. This ensures that when clear real-world images are processed by the student network, they emerge essentially unchanged—a crucial property for practical deployment where the model must handle mixed-quality inputs without degrading satisfactory images.

While adversarial training effectively aligns global distributions, we further introduce our Cluster-Grained Contrastive Learning (CGCL) to address fine-grained structural alignment. The key insight behind CGCL is that meaningful adaptation should occur at the semantic level rather than merely at the instance level. Instead of matching individual degraded images to specific clear counterparts, CGCL organizes the embedding space around semantic clusters, where a degraded image is pulled toward the entire manifold of semantically similar clear images. This approach acknowledges that multiple clear images of similar scenes collectively define the target distribution more robustly than any single exemplar.

As illustrated in Figure 3, CGCL operates through dual pathways: at the feature level, it aligns intermediate representations with clusters of clear image features; at the image level, it ensures the final outputs reside within the natural image manifold. This multi-scale alignment prevents the model from learning superficial statistical matches while encouraging deeper semantic understanding of what constitutes a high-quality restoration.

The complete adaptation objective integrates these complementary components— adversarial distribution matching, identity preservation, and cluster-grained semantic alignment—to comprehensively address the synthetic-to-real domain gap. Through this coordinated approach, the student network evolves from a synthetically competent model to a robust real-world restoration system capable of handling the full complexity of authentic imagery while maintaining a unified parameter set across all degradation types.

### 3.4. Training Losses

The optimization objectives for our two-stage framework are carefully designed to address the distinct challenges of each phase while maintaining conceptual consistency through shared contrastive learning principles.

The knowledge transfer stage employs a composite loss function that guides the student network to assimilate expertise from multiple specialized teachers. The foundation is established by a pixel-wise reconstruction loss:

(1) $$L_{\mathrm{pixel}}=E_{x_{s}^{i}∼X_{S}^{i}}[∥G_{S}\left(x_{s}^{i}\right)-G_{T_{i}}\left(x_{s}^{i}\right){∥}_{1}],\;i=1,\ldots ,k$$

This ensures basic fidelity by minimizing the discrepancy between student and teacher outputs at the pixel level.

The core innovation lies in our IGCL, which operates through dual alignment pathways. We first define a generalized contrastive objective:

(2) $$L_{C}\left(f,f^{+},f^{-}\right)=-\log\frac{\mathrm{sim}\left(\phi \left(f\right),\phi \left(f^{+}\right)\right)}{\mathrm{sim}\left(\phi \left(f\right),\phi \left(f^{+}\right)\right)+\sum_{q=1}^{b}\mathrm{sim}\left(\phi \left(f\right),\phi \left(f_{q}^{-}\right)\right)},$$

where *f*, $f^{+}$, and $f_{j}^{-}$ denote the objective to be optimized, the positive sample, and the negative sample, respectively. *b* denotes the number of negative samples, which is usually equal to the batch size. $\mathrm{sim}\left(u,v\right)=\exp\left(\frac{u^{T}v}{∥u∥∥v∥\tau }\right)$ denotes the similarity between two normalized feature vectors. τ denotes a scalar temperature parameter. ϕ() denotes a feature extraction operation by the VGG-19 [61].

The instance-grained losses are then specified as:

(3) $$L_{fg}=L_{C}\left(fs_{s_{i}},ft_{s_{i}},{\left\{{\left\{fs_{s_{i}}^{q}\right\}}_{i=1}^{k}\right\}}_{q=1}^{b}\right),i=1,2\ldots k,$$

(4) $$L_{ig}=L_{C}\left(xs_{s_{i}},xt_{s_{i}},{\left\{{\left\{x_{s_{i}}^{q}\right\}}_{i=1}^{k}\right\}}_{q=1}^{b}\right),i=1,2\ldots k,$$

where $fs_{s_{i}}$ and $xs_{s_{i}}$ are intermediate features and restored image from the student, the positive samples $ft_{s_{i}}$ and $xt_{s_{i}}$ are intermediate features and restored image from the teacher, the negative samples ${\left\{{\left\{fs_{s_{i}}^{q}\right\}}_{i=1}^{k}\right\}}_{q=1}^{b}$ and ${\left\{{\left\{x_{s_{i}}^{q}\right\}}_{i=1}^{k}\right\}}_{q=1}^{b}$ are a batch of intermediate features from the student and a batch of synthetic degraded images, respectively.

The complete knowledge transfer objective integrates these components:

(5) $$L_{m}=L_{ig}+\alpha_{1}L_{fg},$$

(6) $$L_{kt}=L_{pixel}+\alpha_{2}L_{m},$$

where $\alpha_{1}$ and $\alpha_{2}$ are trade-off weights.

The domain adaptation stage introduces objectives specifically designed to bridge the synthetic–real domain gap. The adversarial component employs a generative adversarial loss:

(7) $$L_{gan}=E_{x_{r}^{i}∼X_{R}^{i}}\left[D_{S}\left(G_{S}\left(x_{r}^{i}\right)\right)\right]+E_{x_{c}∼X_{C}}\left[D_{S}\left(G_{S}\left(x_{c}\right)\right)-1\right].$$

This encourages the student network to produce outputs that are indistinguishable from real clear images.

To preserve content integrity, we incorporate an identity preservation loss:

(8) $$L_{idt}=E_{x_{c}∼X_{C}}\left[{∥G_{S}\left(x_{c}\right)-x_{c}∥}_{1}\right].$$

The CGCL component addresses the lack of strongly correlated positives in real-world data by employing cluster-level alignment:

(9) $$\begin{array}{c} L_{EC}\left(f,f^{+},f^{-}\right)=-\log\frac{\sum_{p=1}^{b}\mathrm{sim}\left(\phi \left(f\right),\phi \left(f_{p}^{+}\right)\right)}{\sum_{p=1}^{b}\mathrm{sim}\left(\phi \left(f\right),\phi \left(f_{p}^{+}\right)\right)+\sum_{q=1}^{b}\mathrm{sim}\left(\phi \left(f\right),\phi \left(f_{q}^{-}\right)\right)}, \end{array}$$

The cluster-grained losses are then defined as:

(10) $$L_{dfg}=L_{EC}\left(fs_{r_{i}},{\left\{fs_{c}^{p}\right\}}_{p=1}^{b},{\left\{{\left\{fs_{r_{i}}^{q}\right\}}_{i=1}^{k}\right\}}_{q=1}^{b}\right),i=1,2\ldots k,$$

(11) $$L_{dig}=L_{EC}\left(xs_{r_{i}},{\left\{xs_{c}^{p}\right\}}_{p=1}^{b},{\left\{{\left\{x_{r_{i}}^{q}\right\}}_{i=1}^{k}\right\}}_{q=1}^{b}\right),i=1,2\ldots k,$$

where $fs_{r_{i}}$ and $xs_{r_{i}}$ are intermediate features and restored image of the real-world degraded image, the positive samples ${\left\{fs_{c}^{p}\right\}}_{p=1}^{b}$ and ${\left\{xs_{c}^{p}\right\}}_{p=1}^{b}$ are intermediate features and restored images of a batch of real-world clear images, the negative samples ${\left\{{\left\{fs_{r_{i}}^{q}\right\}}_{i=1}^{k}\right\}}_{q=1}^{b}$ and ${\left\{{\left\{x_{r_{i}}^{q}\right\}}_{i=1}^{k}\right\}}_{q=1}^{b}$ are a batch of intermediate features of the real-world degraded images and a batch of real-world degraded images, respectively.

The complete domain adaptation objective combines these elements:

(12) $$L_{dm}=L_{dig}+\lambda_{1}L_{dfg},$$

(13) $$L_{da}=L_{gan}+\lambda_{2}\left(L_{idt}+L_{dm}\right),$$

where $\lambda_{1}$ and $\lambda_{2}$ are trade-off weights.

This unified loss formulation provides a comprehensive optimization framework that progressively transitions from knowledge assimilation to domain adaptation while maintaining consistent contrastive learning principles throughout both stages.

## 4. Experiments

In this section, we present the experimental setup and results to evaluate the effectiveness of our proposed framework. We implement our framework based on MPR-Net [22] and conduct experiments on both synthetic and real-world degradation removal datasets. We compare our method against several state-of-the-art approaches and evaluate the visual quality and performance using commonly adopted metrics. Additionally, we perform two ablation studies to demonstrate the effectiveness of our proposed loss function and framework on both synthetic and real-world degradation removal datasets.

### 4.1. Implementation Details

#### 4.1.1. Datasets

To comprehensively evaluate the proposed Uni-Removal method, we employ a series of widely recognized benchmark datasets for image restoration tasks, including dehazing, deraining, deblurring, and denoising. A key principle of our experimental design is the structured use of data: we leverage synthetic datasets for their reliable ground-truth in the initial knowledge transfer stage, and transition to real-world datasets to validate the model’s practical generalization in the domain adaptation stage and final testing. The specifications and usage of all datasets are summarized in Table 3.

For single image dehazing, we utilize the RESIDE dataset [62], which comprises five subsets. We employ the synthetic Outdoor Training Set (OTS) for first-stage training, while the Synthetic Object Testing Set (SOTS) is used for corresponding validation and ablation studies. The model is then fine-tuned and evaluated on real-world images using the Unannotated Real Hazy Images (URHI) and Real Task-driven Testing Set (RTTS) subsets.

For single image deraining, we employ the Rain1400 dataset [63] for training and validating the first stage of our model. To evaluate the generalization capability of our final model, we fine-tune and test it on the SPatial Attentive (SPA) dataset [32], which consists of real rainy images with diverse and complex rain streak patterns.

For single image deblurring, the synthetic GoPro dataset [64] is used for training and validating the first stage. We then transition to real-world data by fine-tuning our model on the RealBlur dataset [65], which comprises two subsets RealBlur-J and RealBlur-R. The final evaluation is conducted on both RealBlur-J and RealBlur-R to assess robustness.

For single image denoising, we extend our evaluation to the Smartphone Image Denoising Dataset (SIDD) [66] benchmark. It is used exclusively for testing the denoising performance of our pre-trained model, demonstrating its zero-shot generalization ability.

All subsequent experimental results are obtained based on the above datasets. When training Multiple Adverse Weather Removal (MAWR) [27] and our Uni-Removal, we used a mixture of OTS [62], Rain1400 [63], and GoPro [64], and when fine-tuning Uni-Removal, we used a mixture of URHI [62], SPA [32], and BLUR-J [65]. In the training phase, all images are randomly cropped into patches of size 128×128. The pixel values of the patches are normalized to the range of −1 to 1.

#### 4.1.2. Training Details

Our experimental framework was deployed on a computational environment consisting of an NVIDIA GeForce RTX 3090 GPU with CUDA 11.6 and cuDNN 8.5.0, operating under Ubuntu 20.04 LTS. All models were implemented using PyTorch 1.10.0 and optimized with the ADAM (Adaptive Moment Estimation) algorithm using a consistent batch size of 16 across experiments. The fvcore library was employed for profiling computational complexity metrics including FLOPs and parameter counts.

The training process follows a carefully designed protocol to ensure optimal knowledge transfer and domain adaptation. In the knowledge transfer stage, the student network undergoes comprehensive training for 200 epochs with a learning rate of $2\times 10^{-5}$. The optimizer configuration uses momentum parameters $\beta_{1}=0.9$ and $\beta_{2}=0.999$ to maintain stable gradient updates. The contrastive learning components are balanced using trade-off weights $\alpha_{1}=0.5$ and $\alpha_{2}=0.1$, with $\alpha_{1}$ undergoing exponential decay at a rate of 0.99 per epoch to progressively reduce the influence of feature-level alignment as training advances.

In the domain adaptation stage, the pre-trained student network undergoes fine-tuning for 40 epochs with a reduced learning rate of $5\times 10^{-6}$ to facilitate precise parameter adjustments. The optimizer momentum is reconfigured with $\beta_{1}=0.5$ and $\beta_{2}=0.999$ to accommodate the different optimization landscape of adversarial training. The adaptation process employs trade-off weights $\lambda_{1}=0.5$ and $\lambda_{2}=0.1$, with $\lambda_{1}$ similarly decaying at 0.99 per epoch to gradually emphasize image-level alignment over feature-level constraints.

A critical hyperparameter shared across both stages is the temperature setting $\tau =1\times 10^{-6}$ for our contrastive losses, which sharpens the focus on challenging negative samples and enhances fine-grained discrimination capability.

This configuration establishes a reproducible experimental foundation that ensures fair comparison and facilitates future research in unified image restoration.

### 4.2. Comparisons with State-of-the-Art Methods

In this section, we conduct a comprehensive evaluation of Uni-Removal against leading contemporary approaches across diverse real-world degradation benchmarks. We evaluate the performance both qualitatively and quantitatively, using commonly adopted metrics. For the sake of fairness, we prefer to use the codes and the trained models provided by the authors.

#### 4.2.1. Visual Quality Comparison

**Figure 4:**
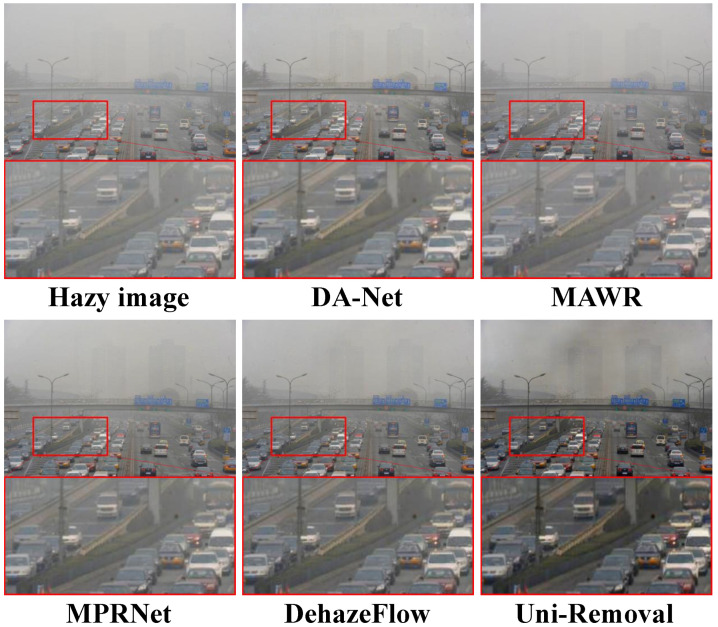
Comparison of dehazing results on real-world hazy images from RTTS [62]. Uni-Removal demonstrates superior performance in effectively removing haze while avoiding the introduction of artifacts, surpassing other existing methods.

To evaluate the visual quality of Uni-Removal, we conducted experiments on the real-world haze removal dataset RTTS [62], which is a subset of the RESIDE dataset [62]. We compared the results of Uni-Removal with six state-of-the-art dehazing methods: Domain Adaptation Network (DA-Net) [38], MAWR [27], MPR-Net [22] (the backbone), DehazeFlow [17], Weather-General and Weather-Specific (WGWS) [67] and Patil et al. [28]. The compared methods encompass specialized dehazing networks, unified models with parameter switching, and recent All-in-One approaches. The results are shown in Figure 4.

From the visual comparisons in Figure 4, it can be observed that DA-NET [38] removes a significant amount of haze, but the color of the images is shifted, and some black shadows appear. Models trained exclusively on synthetic data demonstrate limited generalization to real-world haze patterns, with visible residual artifacts and incomplete restoration. In contrast, Uni-Removal generates images with notably reduced haze residue and successfully preserves the background color, texture, and other details, yielding favorable results in terms of visual quality.

**Figure 5:**
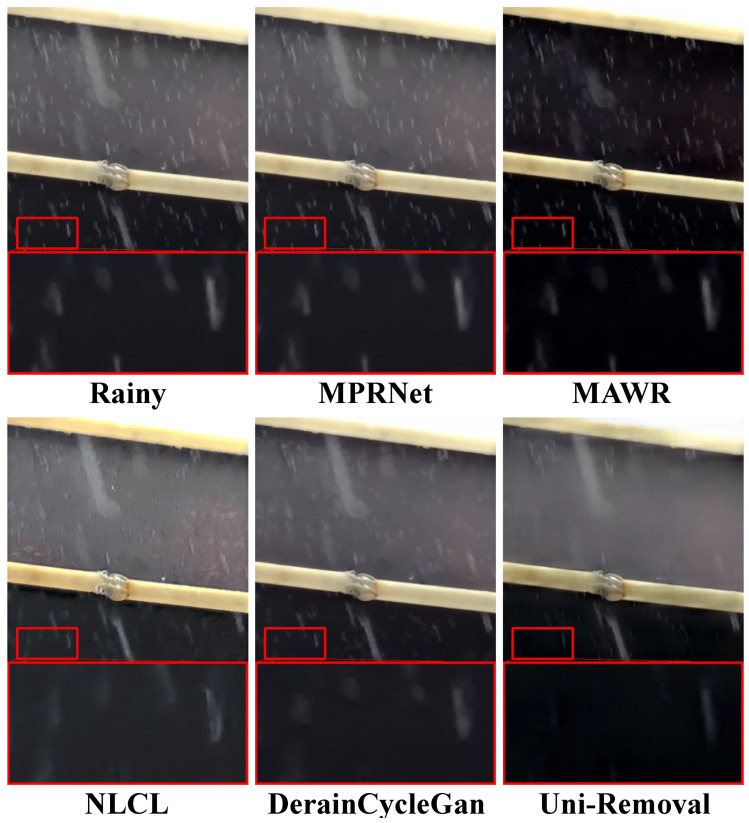
Comparison of deraining results on real-world rainy images from SPA [32]. Uni-Removal achieves remarkable rain streak removal performance. It outperforms all other state-of-the-art methods, effectively removing the majority of rain streaks with exceptional quality.

Next, we evaluated the visual quality of Uni-Removal on the real-world rain streaks removal dataset SPA [32]. We compared the results of Uni-Removal with six state-of-the-art deraining methods: MPR-Net [22], MAWR [27], NLCL [56], DerainCycleGan [39], WGWS [67] and Patil et al. [28]. Both NLCL [56] and DerainCycleGan [39] are unsupervised methods trained on real-world rain streaks removal datasets. The results are shown in Figure 5.

As illustrated in Figure 5, compared to MPR-Net [22] and Patil et al. [28], unsupervised domain adaptation-based method DerainCycleGan [39] demonstrates better performance in effectively removing rain streaks from real-world images. Although MAWR [27] removes the majority of the rain streaks, the resulting images are generally too dark, leading to poor visual quality. In contrast, Uni-Removal achieves more complete rain streak suppression while better preserving underlying image structures and natural color rendition.

**Figure 6:**
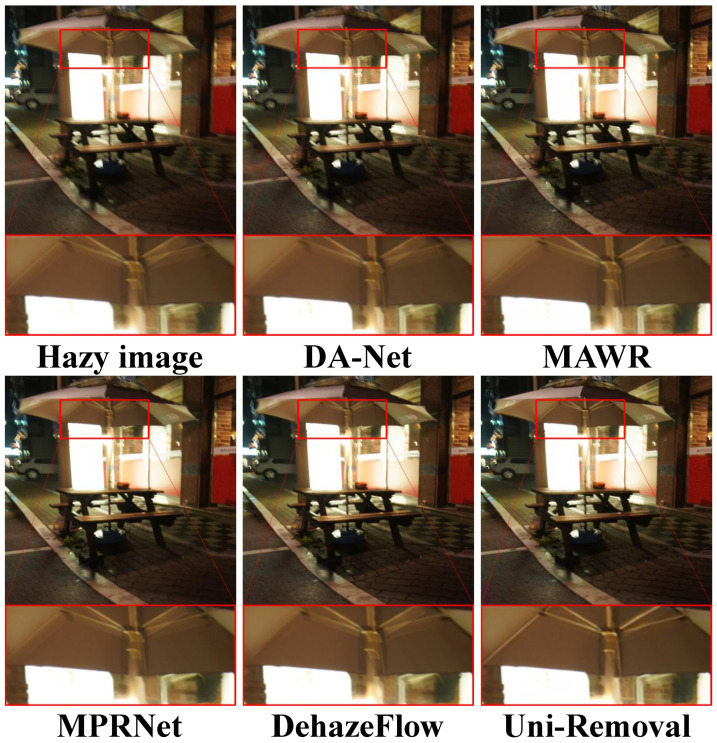
Comparison of deblurring results on real-world blurry images from RealBlur-J [65]. Uni-Removal demonstrates its capability to deliver superior deblurring results, particularly in text scenes. The Korean text on the signboard is a place name.

Lastly, we assessed the visual quality of Uni-Removal on the real-world blur removal dataset RealBlur-J [65]. We compared the results of Uni-Removal with six state-of-the-art deblurring methods: MAWR [27], XYDeblur [68], MPR-Net [22], DeblurGan-v2 [37], WGWS [67] and Patil et al. [28]. XYDeblur [68] and DeblurGan-v2 [37] are state-of-the-art supervised and unsupervised deblurring methods, respectively. The results are shown in Figure 6.

Consistent with trends observed in dehazing and deraining, unsupervised adaptation proves advantageous, with DeblurGan-v2 [37] outperforming purely supervised approaches. Furthermore, Uni-Removal outperforms all the supervised blur removal methods and achieves comparable or improved visual quality to DeblurGan-v2 [37] in terms of visual quality.

Collectively, these qualitative comparisons establish Uni-Removal’s advantage over both specialized unified architectures and contemporary All-in-One solutions. Furthermore, Uni-Removal exhibits promising results in terms of visual quality when compared to task-specific state-of-the-art semi-supervised and unsupervised methods.

#### 4.2.2. No-Reference Image Quality Assessment

We perform rigorous quantitative assessment using established no-reference metrics to objectively measure restoration quality across three challenging real-world benchmarks: RTTS [62] (dehazing), SPA [32] (deraining), and RealBlur-J [65] (deblurring).

Due to the absence of ground-truth clear images for real-world degraded test sets, full-reference metrics like PSNR and SSIM are not applicable. Therefore, we employed two widely adopted no-reference image quality assessment (NR-IQA) metrics: BRISQUE [69] and PIQE [70], where lower values indicate higher perceptual quality.

These metrics complement each other by evaluating different aspects of perceptual quality—BRISQUE focuses on naturalness preservation while PIQE assesses localized artifacts. We calculated the results using the official MATLAB functions provided for these two evaluators. Lower values of these indicators indicate higher image quality. The results are summarized in Table 4, Table 5 and Table 6.

As shown in Table 4, Uni-Removal demonstrates superior and consistent dehazing performance across both real-world benchmarks. On the RTTS dataset, our method achieves the best scores on both BRISQUE and PIQE, with notable improvements of 0.67 and 6.20 over the second-best method, respectively. This significant margin, particularly in PIQE, underscores its exceptional capability in producing perceptually realistic results. The performance generalizes robustly to the more challenging URHI dataset, where Uni-Removal attains the lowest PIQE score (29.64) and a highly competitive BRISQUE score, lagging by only 0.43 from the best result. The consistent superiority across both constrained (RTTS) and unconstrained (URHI) haze scenarios demonstrates our framework’s adaptability to varying real-world conditions. The results confirm that our model successfully bridges the synthetic-to-real domain gap without overfitting to a specific data distribution.

For deraining evaluation on SPA (Table 5), Uni-Removal achieves the most favorable scores on both metrics, reducing BRISQUE by 1.48 and PIQE by 5.79 compared to the nearest competitor. Similarly, for deblurring on RealBlur-J (Table 6), it achieves a BRISQUE improvement of 1.01, suggesting effective blur removal without introducing noticeable artifacts.

Additionally, Table 6 demonstrates the consistent and superior deblurring capability of Uni-Removal across both RealBlur-J and RealBlur-R datasets. On RealBlur-J, our method achieves the best BRISQUE score (39.97), representing a substantial improvement of 1.01 over the second-best method, confirming its effectiveness in removing JPEG-compressed blur without introducing artifacts. While DeblurGan-v2 achieves the best PIQE on this subset, our method maintains a competitive PIQE score. More importantly, the advantage of our framework becomes particularly evident on the more challenging RealBlur-R dataset, which contains raw sensor data with complex blur patterns. Here, Uni-Removal achieves the best performance on both BRISQUE (36.61) and PIQE (44.37), outperforming the second-best method by 2.24 and 4.97 points, respectively. The performance advantage is particularly pronounced on RealBlur-R’s raw sensor data, indicating superior handling of complex, unprocessed blur patterns beyond conventional JPEG artifacts.

These quantitative findings corroborate the qualitative observations, collectively establishing Uni-Removal’s comprehensive capability in real-world degradation removal.

### 4.3. Cross-Task Validation: Image Denoising

To further validate the generalization capability and robustness of our proposed Uni-Removal framework beyond the degradations it was primarily designed for, we evaluate its performance on the challenging image denoising task using the SIDD benchmark, comparing it against several state-of-the-art methods including both specialized denoisers and unified restoration models.

The quantitative results are summarized in Table 7. It is noteworthy that, without any architectural modification or task-specific tuning for denoising, our unified model achieves highly competitive performance, attaining a PSNR of 39.83 dB and an SSIM of 0.958. This places our method as a strong contender among the top-performing models, closely approaching the top PSNR value (within 0.12 dB) and matching the best SSIM score. This demonstrates that the representation and restoration capability learned by our framework through multi-task knowledge transfer and real-world domain adaptation is effectively transferable, yielding competitive results even on an unseen degradation type like noise.

**Figure 7:**
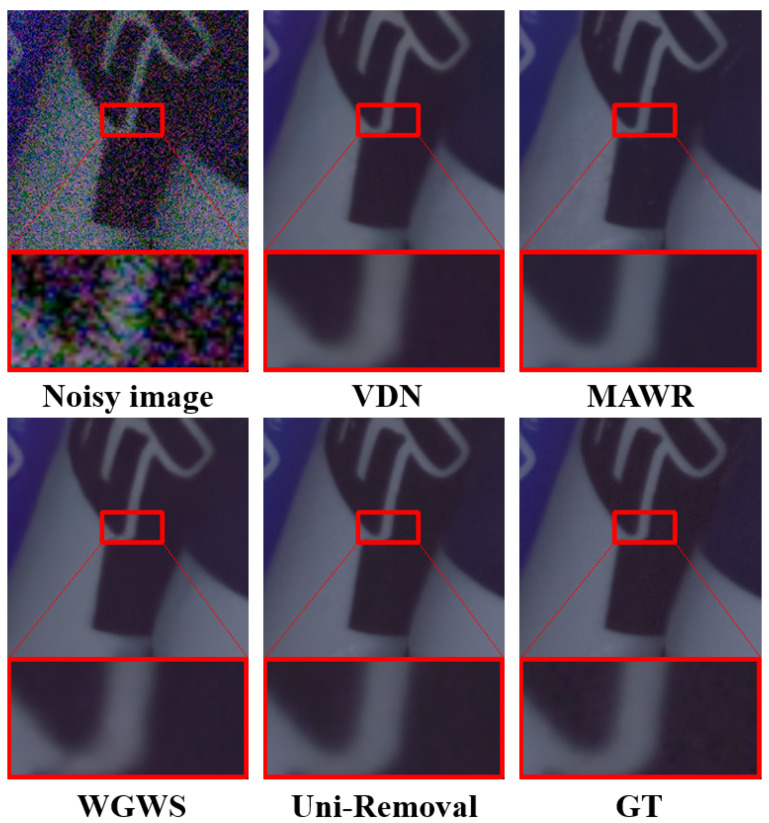
Denoising results on the SIDD benchmark [66] as a cross-task validation. Notably, without any architectural modification for denoising, our unified Uni-Removal framework successfully removes noise and recovers a clear image, demonstrating its exceptional generalization capability.

Qualitative results, as visually attested in Figure 7, corroborate the quantitative findings. Uni-Removal suppresses noise while meticulously preserving fine textures and edges, producing clean and natural-looking images. The highly competitive performance in this cross-task validation underscores the robustness and versatility of our semi-supervised framework, confirming its efficacy as a general-purpose solution for real-world image restoration.

### 4.4. Object Detection Evaluation

A primary motivation for image restoration is to enable robust performance in high-level computer vision tasks. To quantitatively assess the practical utility of our dehazing method, we evaluate the object detection performance on images restored by different methods using the YOLOv8 [73] model. The results are summarized in Table 8.

Quantitatively, our Uni-Removal achieves a mean Average Precision (mAP) of 68.72, which represents the best performance among all compared methods and provides a substantial gain of 4.36 mAP over the detection results on the original hazy images. This superior score indicates that the cleaner and more detailed images produced by our framework provide a higher quality input for the detection network.

**Figure 8:**
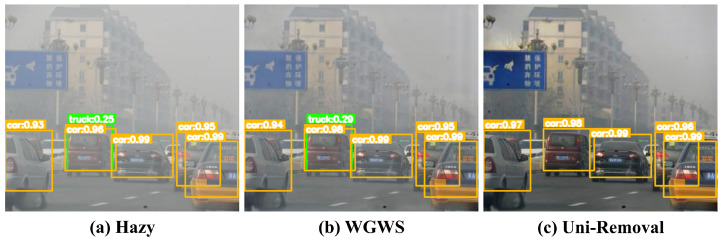
Object Detection on (**a**) hazy and restored images from (**b**) WGWS [67] and (**c**) Uni-Removal using YOLOv8 [73]. The Chinese texts on the traffic sign are place names.

Qualitatively, Figure 8 provides a compelling visual explanation for this performance gain. In (a), the object detector struggles with the hazy input, producing a redundant detection box for a truck that heavily overlaps with a car, indicating low localization confidence. While the result from WGWS (b) shows improved visibility, residual haze still degrades the detection clarity, resulting in the same redundant box. In contrast, our method (c) successfully removes the haze that caused the ambiguity, yielding the cleanest detection result where the redundant truck box is eliminated, and the confidence scores for all correctly detected objects are the highest. This case clearly demonstrates that our restoration output not only enhances visual quality but also provides a more reliable and semantically coherent visual representation for downstream autonomous systems by reducing prediction uncertainty.

### 4.5. Ablation Study

We perform systematic ablation analyses to quantify the individual and collective contributions of our core technical innovations—the IGCL, CGCL, and the overall domain adaptation strategy—across both synthetic and real-world evaluation environments.

The knowledge transfer stage is first analyzed to isolate the impact of our proposed contrastive learning components on synthetic data restoration. We compared three combinations in the knowledge transfer stage using three synthetic degradation datasets: SOTS [62] (dehazing), Rain1400 [63] (deraining), and GoPro [64] (deblurring). We employed PSNR and SSIM as quantitative evaluation metrics. The results are presented in Table 9.

Table 9 indicates that both the image-grained contrastive learning loss and the feature-grained contrastive learning loss improve the effectiveness of the knowledge transfer stage across all three datasets. The complete knowledge transfer model shows PSNR improvements of 0.291 dB, 0.210 dB, and 1.499 dB (and SSIM improvements of 0.007, 0.004, and 0.041) on the SOTS, Rain1400, and GoPro datasets, respectively, compared to the base model. Moreover, the complete knowledge transfer model shows minimal performance degradation compared to the teacher network trained on task-specific datasets.

**Figure 9:**
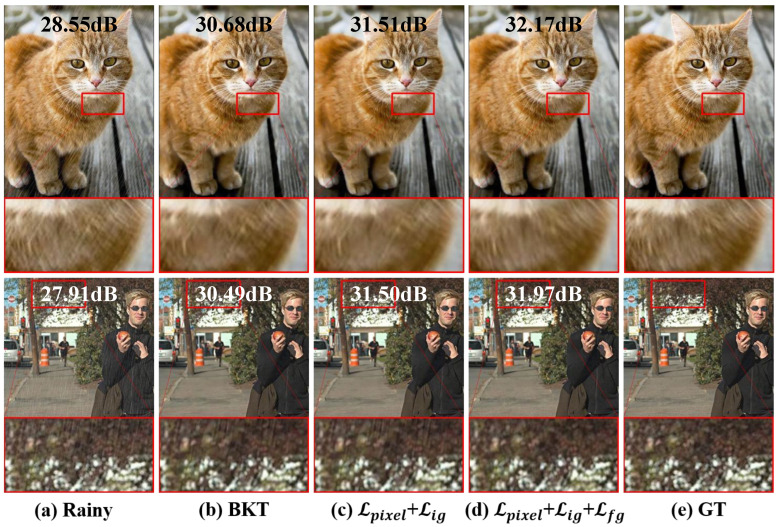
Ablation study of the IGCL in the knowledge transfer stage using synthetic rainy images. (**a**,**e**) represent the rainy images and their respective ground truth from Rain1400 [63]. ‘BKT’ refers to base knowledge transfer training with only the pixel-level loss $L_{pixel}$. To facilitate a quantitative assessment of the improvements, the PSNR↑ (dB) value of each restored image is annotated directly on the figure. Subfigures (**b**–**d**) correspond to the configurations in Table 9, while subfigure (**e**) represents the ground truth.

Figure 9 provides visual results for the aforementioned combinations. Visual analysis in Figure 9 shows progressive artifact reduction: the baseline retains visible rain streaks, while successive addition of contrastive components yields cleaner outputs with improved structural fidelity. The background image restored by the complete knowledge transfer not only exhibits the fewest residual rain streaks but also closely resembles the ground truth in terms of clarity and texture.

For real-world performance, we evaluate the domain adaptation module’s efficacy in bridging the synthetic-to-real gap, with particular focus on the CGCL mechanism. We compared four combinations in the domain adaptation stage using three real-world degradation datasets: RTTS [62] (dehazing), SPA [32] (deraining), and BLUR-J [65] (deblurring). For quantitative comparison, we utilized BRISQUE [69] and PIQE [70] as evaluation indicators. The results are presented in Table 10.

As presented in Table 10, the knowledge transfer model without domain adaptation exhibits poor performance on real-world degradation removal tasks. However, with domain adaptation, notable improvements are observed, with enhancements of 5.94, 4.95, 2.56 and 14.73, 8.69, 10.92 in terms of BRISQUE and PIQE on the three real-world datasets, respectively. Additionally, both the cluster image-grained contrastive learning loss and the cluster feature-grained contrastive learning loss contribute to the quality improvement of restored background images, including naturalness, color, distortion, and other factors. Compared to the base domain adaptation model, the full Uni-Removal demonstrates further improvements of 3.20, 2.97, 1.54 and 5.35, 4.79, 4.13 in BRISQUE and PIQE on the three real-world datasets, respectively.

**Figure 10:**
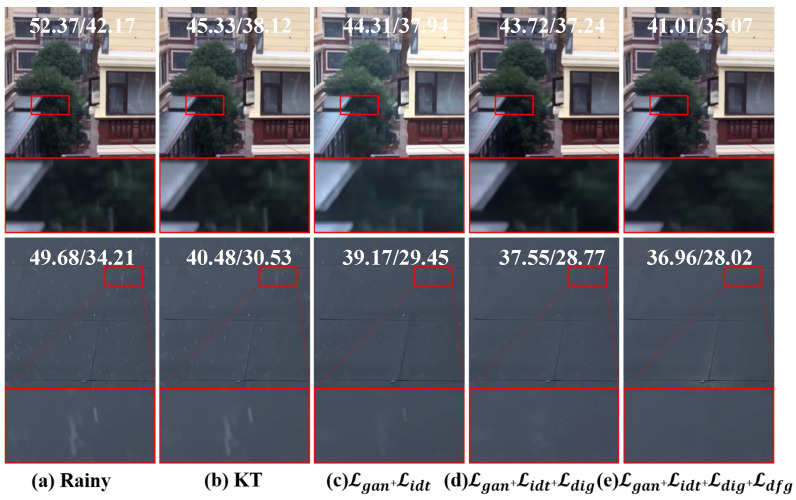
Ablation study of the domain adaptation stage and the CGCL using real-world rainy images. (**a**) displays rainy images from SPA [32]. ‘KT’ represents knowledge transfer with the IGCL loss. To facilitate a quantitative assessment of the improvements, the BRISQUE↓/PIQE↓ value of each restored image is annotated directly on the figure. Subfigures (**b**–**e**) correspond to the configurations in Table 10.

Figure 10 illustrates the visual outcomes obtained from the four aforementioned combinations. The performance of the knowledge transfer model alone struggles when it comes to eliminating rain streaks in real-world images. However, incorporating the domain adaptation training stage significantly enhances the model’s ability to derain such images effectively. Moreover, the inclusion of the cluster image-grained contrastive learning loss and the cluster feature-grained contrastive learning loss further contributes to improving the overall quality of the restored images. The complete Uni-Removal configuration produces the most visually convincing results, effectively eliminating degradation artifacts while maintaining natural image characteristics. Collectively, these ablation results across six benchmarks provide robust validation of our architectural choices and demonstrate the critical role each component plays in achieving state-of-the-art restoration performance.

### 4.6. Complexity Analyses

Beyond restoration quality, practical deployment requires careful consideration of computational demands. We benchmark Uni-Removal against contemporary All-in-One models [27,28,67] across multiple efficiency metrics, with results detailed in Table 11.

The analysis reveals that our framework achieves an exceptional balance between performance and efficiency. With merely 3.6M parameters, Uni-Removal requires 3.2× fewer parameters than the most parameter-efficient competitor (Patil et al. [28]), significantly reducing memory footprint and storage requirements. This compact design is complemented by computational economy, as evidenced by a low FLOP count that underscores operational efficiency. Inference speed, critical for real-world applications, is particularly noteworthy. Uni-Removal processes a 512×512 resolution image in just 0.0728 s, outperforming all compared methods. This rapid execution enables seamless integration into time-sensitive applications without compromising restoration quality.

The combination of minimal parameterization, computational frugality, and accelerated inference establishes Uni-Removal as a compelling solution for resource-constrained environments, including mobile platforms and edge computing devices where both memory and processing capabilities are limited.

### 4.7. Hyper-Parameters Analysis

Hyper-parameters analysis is also performed on a mixed evaluation set comprising dehazing, deraining, and deblurring. The analysis of trade-off weights and batch size is summarized in Table 12. The results indicate that the configuration with $\alpha_{1}$ = 0.5, $\alpha_{2}$ = 0.1 and a batch size of 16 yields the best performance across both knowledge transfer and domain adaptation stages. This aligns with the expectation that a larger batch size benefits contrastive learning by providing more stable and diverse negatives.

### 4.8. Limitations and Failure Case Analysis

**Figure 11:**
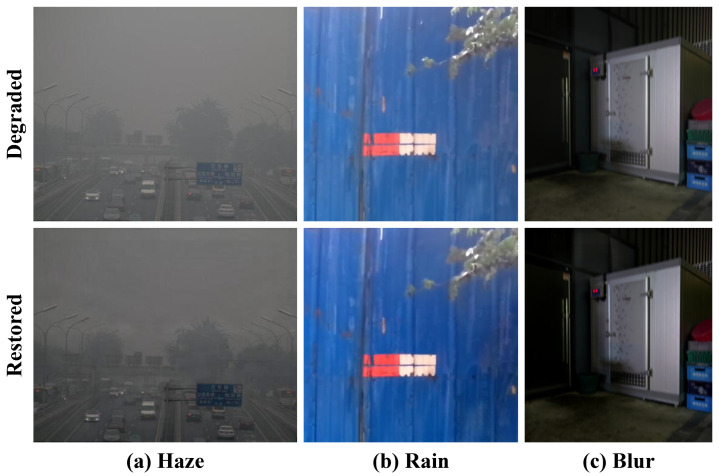
Typical failure cases of Uni-Removal under challenging low-light and severe degradation conditions. The model exhibits limited restoration efficacy for (**a**) dense haze, (**b**) heavy rain, and (**c**) blur when the scenes are extremely dark or poorly illuminated.

While Uni-Removal demonstrates robust performance across a variety of scenarios, a deliberate analysis of its failure modes offers valuable insights into its current limitations and paves the way for future improvements. As illustrated in Figure 11, the model’s restoration efficacy diminishes under extremely challenging environmental conditions, particularly in scenes with severely low illumination coupled with strong degradations. In these cases, such as dense haze at dusk (a), heavy rainfall at night (b), or significant blur in dark environments (c), the inherent lack of visual information and low signal-to-noise ratio presents a fundamental challenge. The framework struggles to sufficiently remove the degradations, as the core visual cues required for both knowledge transfer and domain adaptation are critically compromised. This observation underscores a key limitation of our current approach and highlights that extreme low-light conditions remain an open challenge for general-purpose restoration models. Addressing this issue may require the explicit integration of low-light enhancement techniques or the curation of specialized training datasets encompassing such extreme cases, which we identify as a promising direction for future work.

## 5. Conclusions

This paper has introduced Uni-Removal, a semi-supervised framework that tackles multi-degradation removal in real-world images through a coordinated two-stage approach. By systematically combining knowledge distillation with domain adaptation, our method provides an effective solution for unified image restoration.

Comprehensive validation shows that Uni-Removal achieves superior performance, substantially outperforming existing methods on real-world benchmarks for dehazing (+0.67 BRISQUE, +6.20 PIQE), deraining (+1.48 BRISQUE, +5.79 PIQE), and deblurring (+1.01 BRISQUE). The framework also demonstrates exceptional generalization, delivering competitive denoising results (39.83 dB PSNR on SIDD) without architectural changes. Most importantly, it offers practical utility by improving object detection accuracy by 4.36 mAP, underscoring its real-world value.

These capabilities make Uni-Removal a compelling solution for practical systems. Its parameter-efficient design is suitable for computational photography on mobile devices, enhancing images captured in adverse conditions. Furthermore, its reliability as a pre-processing module benefits safety-critical applications like autonomous driving and surveillance, where image quality directly impacts performance.

## Figures and Tables

**Table 1:** Comparison of representative All-in-One image restoration methods.

Method	Unification Strategy	Shared Params	Real-World	Key Limitations
MPR-Net [22]	Unified model, different parameters	No	Synthetic	Requires switching parameters for different tasks.
TransWeather [26]	Single transformer model	Yes	Synthetic	Performance drop due to synthetic-to-real domain gap.
Chen et al. [27]	Multiple encoders, shared decoder	Partially	Synthetic	Complex architecture; not fully parameter-efficient.
Patil et al. [28]	Multi-head network	Partially	Synthetic	Relies on accurate degradation-type input.
Uni-Removal	Two-stage semi-supervised framework	Yes	Real-World	Explicitly bridges the synthetic-to-real domain gap with a unified parameter set.

**Table 2:** Comparison of contrastive learning approaches in low-level vision tasks.

Method	Application Scenario	Learning Paradigm	Positive/Negative Strategy	Key Limitation/Focus
SimCLR [49]	High-level Rep. Learning	Self-supervised	Different augmentations of same image	Requires large batch sizes; not designed for restoration.
MoCo [50]	High-level Rep. Learning	Self-supervised	Queue-based dictionary	Focuses on building a large and consistent dictionary.
Park et al. [55]	Image Translation	Supervised (GAN)	Between input and output domains	Focused on style transfer, not degradation removal.
Chen et al. [58]	Unpaired Dehazing	Unsupervised (GAN)	Between hazy and clear domains	Addresses a single task (dehazing) in one stage.
Ye et al. [56]	Deraining	Unsupervised	Non-local self-similarity patches	Focuses on a single task and intra-image relations.
IGCL (Ours)	Multi-task Knowledge Transfer	Supervised	Student vs. Multiple Teacher outputs	Enables unified model learning from specialized teachers.
CGCL (Ours)	Synthetic-to-Real Adaptation	Self-supervised	Degraded image vs. Cluster of clear images	Anchors the model to the manifold of real-world clear images.

**Table 3:** Dataset specifications for experimental comparison.

Dataset	Number	Degradation	Category	Purpose
OTS [62]	3131	haze	synthetic	Training
SOTS [62]	1000	haze	synthetic	Validation
Rain1400 [63]	1400	rain	synthetic	Training/Validation
GoPro [64]	3214	blur	synthetic	Training/Validation
RTTS [62]	4322	haze	real-world	Validation/Test
URHI [62]	5188 + 1501	haze	real-world	Training/Test
SPA [32]	638,492	rain	real-world	Training/Validation/Test
RealBlur-J [65]	980	blur	real-world	Training/Validation/Test
RealBlur-R [65]	980	blur	real-world	Training/Test
SIDD [66]	30,000	noise	real-world	Test

**Table 4:** Quantitative results using NR-IQA metrics on RTTS [62] and URHI [62]. Lower BRISQUE/PIQE indicate better performance. The best and second-best results are highlighted in bold and underline, respectively. The symbol ↓ indicates lower is better.

Dataset	RTTS		URHI	
Method	BRISQUE [69]↓	PIQE [70]↓	BRISQUE [69]↓	PIQE [70]↓
Hazy	37.01	51.25	34.25	44.48
DA-Net [38]	32.46	50.79	31.52	36.28
MAWR [27]	27.12	45.63	28.86	33.64
MPR-Net [22]	30.24	43.51	29.66	39.25
DehazeFlow [17]	26.06	38.88	27.73	32.77
WGWS [67]	30.32	37.07	30.26	31.92
Patil et al. [28]	25.70	42.00	**26.48**	33.04
Uni-Removal (ours)	**25.03**	**30.87**	26.91	**29.64**

**Table 5:** Quantitative results using NR-IQA metrics on SPA [32]. Lower BRISQUE/PIQE indicate better performance. The best and second-best results are highlighted in bold and underline, respectively. The symbol ↓ indicates lower is better.

Method	BRISQUE [69]↓	PIQE [70]↓
Rainy	68.03	44.39
MPR-Net [22]	76.63	46.76
MAWR [27]	54.72	44.65
NLCL [56]	56.95	43.29
DerainCycleGan [39]	59.28	41.58
WGWS [67]	41.97	66.66
Patil et al. [28]	45.43	50.25
Uni-Removal (ours)	**40.49**	**35.79**

**Table 6:** Quantitative results using NR-IQA metrics on RealBlur [65]. Lower BRISQUE/PIQE indicate better performance. The best and second-best results are highlighted in bold and underline, respectively. The symbol ↓ indicates lower is better.

Dataset	RealBlur-J		RealBlur-R	
Method	BRISQUE [69]↓	PIQE [70]↓	BRISQUE [69]↓	PIQE [70]↓
Blurry	56.41	46.86	46.52	74.43
MAWR [27]	44.92	43.64	43.67	57.64
XYDeblur [68]	70.26	38.90	44.21	61.37
MPR-Net [22]	64.64	36.17	40.06	52.94
DeblurGan-v2 [37]	49.76	**32.59**	38.85	50.77
WGWS [67]	40.98	48.75	39.43	55.37
Patil et al. [28]	42.48	43.84	40.17	49.34
Uni-Removal (ours)	**39.97**	34.52	**36.61**	**44.37**

**Table 7:** Quantitative results on SIDD [66]. Higher PSNR/SSIM indicate better performance. The best and second-best results are highlighted in bold and underline, respectively. The symbol ↑ indicates higher is better.

Method	PSNR↑	SSIM↑
Noisy	23.66	0.328
VDN [71]	39.28	0.956
DeamNet [72]	39.47	0.957
MAWR [27]	39.55	0.956
MPR-Net [22]	39.71	0.958
WGWS [67]	**39.95**	0.957
Patil et al. [28]	39.74	**0.959**
Uni-Removal (ours)	39.83	0.958

**Table 8:** Object detection results on RTTS [62]. The best and second-best results are highlighted in bold and underline, respectively.

Method	Hazy	MSBDN [74]	DA-Net [38]	WGWS [67]	Uni-Removal
mAP	64.36	66.74	67.02	68.17	**66.72**

**Table 9:** Ablation study of the IGCL in the knowledge transfer stage on three synthetic datasets (OTS, Rain1400, GoPro) in PSNR↑ and SSIM↑. ‘BKT’ refers to base knowledge transfer training with only the pixel-level loss $L_{pixel}$. The symbol ↑ indicates higher is better.

Dataset	SOTS [62]		Rain1400 [63]		GoPro [64]	
Teacher	31.770	0.980	32.622	0.939	31.059	0.886
BKT	28.784	0.964	32.052	0.924	27.100	0.831
+$L_{ig}$	28.387	0.968	32.141	0.926	28.445	0.868
+$L_{ig}$ +$L_{fg}$	29.075	0.971	32.262	0.928	28.599	0.872

**Table 10:** Ablation study of the domain adaptation stage and the CGCL on three real-world datasets (RTTS, SPA, RealBlur-J) in BRISQUE↓ [69] and PIQE↓ [70]. ‘KT’ represents knowledge transfer with the IGCL. The symbol ↓ indicates lower is better.

Dataset	RTTS [62]		SPA [32]		BLUR-J [65]	
Degraded	37.011	51.254	68.025	44.388	56.414	36.864
KT	34.271	50.955	48.391	49.260	44.062	49.571
+$L_{gan}$ +$L_{idt}$	28.333	36.225	43.445	40.576	41.500	38.651
+$L_{gan}$ +$L_{idt}$ +$L_{dig}$	25.961	31.152	42.057	38.874	40.478	36.196
+$L_{gan}$ +$L_{idt}$ +$L_{dig}$ +$L_{dfg}$	25.029	30.874	40.489	35.786	39.966	34.517

**Table 11:** Comparative analysis of computational complexity and training efficiency. FLOPs and inference time are measured on a 512×512 resolution image.

Model	FLOPs	Param	Training Time	Infer Time
Chen et al. [27]	78.3 G	28.7 M	25.28 h	0.0773 s
WGWS [67]	996.2 G	12.6 M	37.67 h	0.1919 s
Patil et al. [28]	1262.9 G	11.1 M	-	0.1098 s
SDA-Net	564.9 G	3.6 M	17.63 h	0.0728 s

**Table 12:** Impact of trade-off weights ($\alpha_{1}$, $\alpha_{2}$/$\lambda_{1}$, $\lambda_{2}$) and batch size on model performance. Performance is evaluated by PSNR/SSIM on synthetic data (KT) and BRISQUE/PIQE on real-world data (DA). The optimal setting is in bold. The symbols ↑ and ↓ indicate higher is better, and lower is better, respectively.

	$\alpha_{1}$/$\lambda_{1}$	$\alpha_{2}$/$\lambda_{2}$	KT(PSNR↑/SSIM↑)	DA(BRISQUE↓/PIQE↓)
Trade-off weights	1.0	0.2	29.63/0.918	37.01/34.72
	0.5	0.2	29.76/0.920	36.55/34.12
	1.0	0.1	29.72/0.921	36.24/33.65
	0.5	0.1	**29.88**/**0.925**	**35.80**/**33.12**
Batch size	4		29.44/0.919	36.92/34.22
	8		29.67/0.921	36.21/33.94
	16		**29.88**/**0.925**	**35.80**/**33.12**

## Data Availability

The original contributions presented in this study are included in the article. Further inquiries can be directed to the corresponding author.
